# Glucose and fatty acid metabolism in infarcted heart from streptozotocin-induced diabetic rats after 2 weeks of tissue remodeling

**DOI:** 10.1186/s12933-015-0308-y

**Published:** 2015-11-09

**Authors:** Christiane Malfitano, Alcione Lescano de Souza Junior, Mariana Carbonaro, Andressa Bolsoni-Lopes, Diego Figueroa, Leandro Ezequiel de Souza, Kleiton Augusto Santos Silva, Fernanda Consolim-Colombo, Rui Curi, Maria Claudia Irigoyen

**Affiliations:** Hypertension Unit, Heart Institute (InCor), Medical School of University of São Paulo, Av. Eneas de Carvalho Aguiar, 44, 05403-000 Sao Paulo, SP Brazil; Department of Physiology and Biophysics, Institute of Biomedical Sciences, University of Sao Paulo, Sao Paulo, Brazil; Nursing Department, State University of Mato Grosso, Alta Floresta, Brazil; Department of Nephrology, School of Medicine of the Federal, University of Sao Paulo, Sao Paulo, Brazil; Laboratory of Translational Physiology, Universidade Nove de Julho, (UNINOVE), Sao Paulo, Brazil

**Keywords:** Glucose metabolism, Fatty acid metabolism, Diabetic hyperglycemia, Myocardial infarction, Remodeling

## Abstract

**Background:**

The effects of streptozotocin (STZ)-induced diabetes on heart metabolism and function after myocardial infarction (MI) remodelling were investigated in rats.

**Methods:**

Fifteen days after STZ (50 mg/kg b.w. i.v.) injection, MI was induced by surgical occlusion of the left coronary artery. Two weeks after MI induction, contents of glycogen, ATP, free fatty acids and triacylglycerols (TG) and enzyme activities of glycolysis and Krebs cycle (hexokinase, glucose-6-phosphate dehydrogenase, phosphofructokinase, citrate synthase) and expression of carnitine palmitoyl-CoA transferase I (a key enzyme of mitochondrial fatty acid oxidation) were measured in the left ventricle (LV). Plasma glucose, free fatty acids and triacylglycerol levels were determined. Ejection fraction (EF) and shortening fraction (SF) were also measured by echocardiography.

**Results:**

Glycogen and TG contents were increased (p < 0.05) whereas ATP content was decreased in the LV of the non-infarcted diabetic group when compared to the control group (p < 0.05). When compared to infarcted control rats (MI), the diabetic infarcted rats (DI) showed (p < 0.05): increased plasma glucose and TG levels, elevated free fatty acid levels and increased activity of, citrate synthase and decreased ATP levels in the LV. Infarct size was smaller in the DI group when compared to MI rats (p < 0.05), and this was associated with higher EF and SF (p < 0.05).

**Conclusions:**

Systolic function was preserved or recovered more efficiently in the heart from diabetic rats two weeks after MI, possibly due to the high provision of glucose and free fatty acids from both plasma and heart glycogen and triacylglycerol stores.

## Background

Diabetic patients often present cardiomyopathy and increased risk of myocardial infarction, both of which are associated with a high rate of mortality [1]. However, experimental studies have demonstrated that the hyperglycemia in diabetic animals promote changes in the heart which reduce the intensity of the injury following a permanent coronary artery ligation. Despite the relevance of these findings, this issue deserves further investigation. The response of uncontrolled hyperglycemia on heart remodelling after ischemia is yet to be fully understood [2–8].

Most studies on heart infarction aimed to investigate the consequences of the diabetic condition in reperfusion. Hearts from diabetic rats have been shown to be more sensitive to ischemia/reperfusion in some studies [9, 10] and to exhibit lower susceptibility in others. In these latter studies, decreased fatty acid (FFA) oxidation [11] and greater glycogenolysis during ischemia [12] are associated with reduced myocardial infarction area [13]. The effects of ischemia/reperfusion on cardioprotection are dependent on shear stress, which induces endothelial dysfunction during myocardial reperfusion [14]. So, the consequences of reperfusion depend on duration of the preceding ischemia, the hyperglycemic state, and [15] on the rate of glucose and fatty acid oxidation [16, 17]. Studies involving permanent ischemia and hyperglycemia on cardioprotection have shown conflicting results.

Exposure to a high glucose medium or diabetes for short periods of time has been found to protect the heart against permanent ischemia. This may be due to the improved ventricular function, and reduced infarcted area and mortality reported for diabetic rats under ischemic heart injury [7, 8, 18]. Our group has previously demonstrated that hyperglycemia may promote the survival of heart cells by increasing angiogenesis [7] and/or by decreasing pro-oxidant damage. These findings were associated to improvement in both redox balance and sympathetic control of the heart function [8]. Fifteen days after myocardial infarction, diabetic animals showed reduced infarct size and heart fibrosis area along with improved systolic function, increased expression of programmed cell survival genes, increased use of glucose as fuel (as suggested by high GLUT-1 expression), and reduced intensity of inflammation [7]. However, the association between myocardial infarction and metabolic changes in the heart induced by diabetes needs to be further investigated.

The main purpose of the present study was to examine the changes in glucose and fatty acid metabolism, and cardiac function in streptozotocin-induced diabetic rats during tissue remodeling after myocardial infarction. The following measurements were carried out in the blood: FFA, triacylglycerol (TG) and glucose levels. The following metabolic parameters were measured in the heart: levels of FFA, TG, ATP, and glycogen and activities of enzymes of glycolysis and Krebs cycle (hexokinase, glucose-6-phosphate dehydrogenase, phosphofructokinase, citrate synthase) and expression of carnitine palmitoyl-CoA transferase I (a key enzyme of fatty acid oxidation). The hypothesis of the present study was that the diabetic state provides a more favorable tissue remodeling condition and preserves heart function after a MI through changes in fatty acid and glucose metabolism in the heart. The protocol of ischemia induced by permanent coronary ligation without reperfusion was chosen in order to: (1) exclude the jeopardizing effects of reperfusion *per se*, such as shear stress and generation of reactive oxygen species (ROS), (2) avoid the combination of other complications of diabetes, such as the arteriosclerosis and reduced heart blood flow and (3) examine tissue remodeling after injury as an attempt to establish new therapeutic approaches in the management of patients who suffered an ischemic heart event.

## Methods

### Animals

Male Wistar rats (250–280 g) were obtained from the Animal Facility at the Medical School of the University of São Paulo, São Paulo, Brazil. The rats received standard laboratory chow and water ad libitum and were housed in cages containing four to five animals at 22 °C in a 12-h dark-light cycle. All experimental procedures were conducted according to the Guidelines for the Use and Care of Animals Research issued by the National Institute of Health, and complied with the ARRIVE guidelines for reporting animal research [19]. The protocol used was approved by the Ethics in Research Committee of the Medical School of the University of São Paulo. All experimental procedures were conducted according to the National Institutes of Health Guidelines for the use and care of animals.

**Fig. 1:**
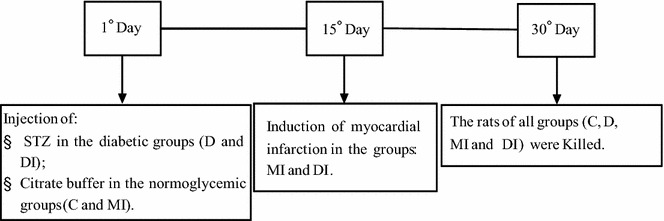
Experimental protocol used to study the effects of myocardial infarction and tissue remodelling on cardiac function and heart glucose and fatty acid metabolism in diabetic and control rats

The rats were randomly assigned to four groups (n = 12 per group): normoglycemic control (C), diabetic (D), normoglycemic myocardial infarcted (MI), and diabetic myocardial infarcted (DI). The diabetic state (D and DI groups) was induced by a single i.v. injection of streptozotocin (STZ, 50 mg/kg b.w; Sigma Chemical Co., St. Louis, MO, USA) dissolved in citrate buffer pH 4.5. This experimental protocol for induction of type I diabetes has been widely used [8, 20–23]. Body weight was weekly evaluated. Blood glucose concentration was measured to confirm diabetes-induced hyperglycemia at the beginning of the 30-day experimental protocol, 24 h after STZ injection (Accu-Check Instant test, Boehringer Mannheim, Germany). The normoglycemic rats (the C and MI groups) received a single injection of citrate buffer. Myocardial infarction was induced in both DI and MI groups, according to the procedure described in the following section. All rats were monitored during 30 days. The schematic representation of the experimental protocol used is depicted in Fig. 1.

Fifteen days after STZ injection or administration of citrate buffer, MI and DI rats were anaesthetized (80 mg/kg b.w. ketamine and 12 mg/kg b.w. xylazine, i.p.) and underwent myocardial infarction by surgical occlusion of the left coronary artery as previously described in several studies [7, 8, 18, 22, 24]. C and D animals underwent sham surgery with no induction of myocardial infarction in order to determine the effects of the surgical procedure itself.

### Echocardiographic measurements in the heart

Echocardiography was performed at the end of the experimental protocol by an observer blinded to group allocation, according to the guidelines of the American Society of Echocardiography. Rats were anaesthetized as described above and images were obtained with a 10-14 mHz linear transducer in a SEQUOIA 512 (ACUSON Corporation, Mountain View, CA, USA) for measurement of systolic function (ejection fraction-EF and fractional shortening-FS), as described in detail elsewhere [23, 24].

MI size was estimated on the basis of akinesis or dyskinesis areas. Regions with systolic shortening were classified as normal, hypokinetic or absent (akinetic), and sections with paradoxical movement were considered dyskinetic. Myocardial infarction size was estimated as the ratio of the infarcted area to the total perimeter of the endocardial border of the LV (%) [25]. To confirm the echocardiographic measurements, MI size was also evaluated by dissecting the fibrous scar from the remaining LV muscle [7].

### Experimental procedure

The animals were euthanized by decapitation after 15 days of the myocardial infarction surgery or 30 days of induction of diabetes (Fig. 1). Plasma and left ventricle were removed and frozen at −70 °C.

### Determination of free fatty acids and triacylglycerols

Plasma levels of FFA were evaluated using NEFA-HR kit (Wako Diagnostics, Richmond, VA, USA). Plasma samples (3 µL) were transferred to a 96-well plate and reagents (200 µL) were added following the manufacturer’s instructions. Measurements of absorbance were carried out at 450 nm. Plasma TG concentrations were determined by enzymatic colorimetric assay following the manufacturer’s protocol (Bioclin^®^, Belo Horizonte, MG, Brazil).

For determination of TG and FFA levels in the LV, the heart tissue was homogenized in chloroform and methanol solution (2:1). The lower phase was then transferred to a new tube and evaporated in a vacuum centrifuge. Lipid pellets were suspended in 100 µL absolute ethanol. The levels of FFA and TG were determined as described above.

### Determination of glycogen content in the LV

Glycogen content of the LV was determined by using the assay described by Keppler and Decker [26]. This method is based on the conversion of glycogen into glucose by amyloglucosidase (Sigma,St. Louis. MO, USA). Glucose from glycogen was quantified using a glucose determination kit (Bioclin) and calculated as mg of glycosyl units/g wet weight of the LV.

### Assays of the enzyme activities

LV protein extracts were sonicated for 30 s, at 4 °C, followed by centrifugation at 1000*g*, at 4 °C, for 20 min. Total protein levels (Bradford method) [27] and maximal enzyme activity were measured in the supernatants.

Hexokinase (HK) activity was determined using the method described by Crabtree and Newsholme [28]. The extraction buffer contained Tris–HCl (50 mM) and EDTA (1 mM) at pH 7.4. The assay buffer consisted of Tris–HCl (75 mM), MgCl_2_ (7.5 mM), EDTA (0.8 mM), KCl (1.5 mM), β-mercaptoethanol (4 mM), NADP^+^(0.4 mM), ATP (2.5 mM), glucose (1 mM), creatine phosphate (10 mM), creatine kinase (1.8 U/mL), glucose-6-phosphate dehydrogenase (1.4 U per mL), and Triton X-100 (0.05 % v/v). The reaction was initiated by the addition of 50 μL glucose (1 mM final concentration) and absorbance measurement at 340 nm during 10 min.

To determine citrate synthase activity we used an extraction buffer containing 0.5 mM Tris–HCl and 1 mM EDTA, pH 7.4, and an assay buffer containing 100 mM), DTNB (0.2 mM), acetyl-CoA (0.1 mM), and Triton X-100 (0.1 % v/v), pH 8.1. The reaction was initiated by the addition of 50 µL oxaloacetic acid (10 mM final concentration) and absorbance measurement at 412 nm during 5 min [29].

### CPT1-M (carnitine palmitoyltransferase I) expression

LV protein was extracted in a buffer containing Tris-base (100 mM, pH 7.5); EDTA 10 mM; sodium fluoride 100 mM; sodium pirophosphate 100 mM; sodium ortovanadate 10 mM; aprotinin 10 mg/mL, leupeptin 1 mg/mL and PMSF 2 mM) and quantified by the Bradford method. Electrophoresis was performed in 7.5 % of slab gel in the presence of sodium dodecyl sulfate (SDS), according to the Laemmli method [30]. Electrotransference was performed for 42 min at a constant voltage (40 V) using a nitrocellulose membrane (Hybond ECL, GE Healthcare, Sweden). The membrane was incubated in a 5 % non-fat dry milk blocking solution for 4 h before overnight incubation at room temperature with polyclonal antibodies at a dilution of 1:1000 (Santa Cruz Biotechnology, CA, USA). The subsequent steps were performed using the streptavidin/phosphatase alkaline system (GE Healthcare, Sweden), and the bands were revealed using NBT/BCIP substrates (Bio Rad Laboratories, CA, USA) [31].

### Determination of ATP level in the heart

LV was homogenized in perchloric acid (0.6 N) and the supernatant was neutralized using potassium bicarbonate aqueous solution (1 M). A commercial kit from Invitrogen (Eugene, Oregon, USA) was used to determine ATP concentration in the supernatant. The method is based on luciferase requirement for ATP in producing light (maximum emission at approximately 560 nm and pH 7.8). ATP levels were calculated as μM and expressed on the basis of wet tissue weight. A kit based on luciferin/luciferase assay has also been recently used by other researchers for determination of ATP levels [32, 33].

### Statistical analysis

Results are presented as mean ± SEM. One-way or two-way ANOVA followed by the Student–Newman–Keuls post hoc test was used to compare the groups. Differences were considered significant at p ≤ 0.05 for all measurements.

## Results

### Indicators of the establishment of diabetic state

After 30 days of STZ injection, body weight was lowered in diabetic rats (D and DI groups) when compared to normoglycemic animals (C and MI groups) (Table 1). Plasma glucose levels were increased in STZ-induced diabetic rats. MI tissue remodelling did not markedly change blood glucose levels either in control or diabetic rats (Table 1). Plasma FFA and triacylglycerol levels were raised in non-infarcted diabetic rats (D group) when compared to the other groups (C, MI and DI). After MI tissue remodelling, plasma FA and triacylglycerol levels were decreased in diabetic rats (DI group) when compared to non- infarcted diabetic rats (D group) (Table 1).

**Table 1:** Measurements of body weight, glucose, free fatty acids (FFA) and triacylglycerol (TG) plasmatic levels after 15 days of myocardial infarction and 30 days of diabetes

Groups	Body weight	Glucose	FFA	TG
C	362 ± 16	87 ± 16	13 ± 1.4	82 ± 6
D	297 ± 16*^†^	433 ± 25*^†^	21 ± 3*^†^	252 ± 44*^†^
MI	378 ± 12	96 ± 3	15 ± 1.7	59 ± 6
DI	305 ± 19*^†^	411 ± 28*^†^	12 ± 0.9^‡^	110 ± 19^‡^

Body weight, Glucose, FFA and TG levels in plasma (mg/dl) of control (C), diabetic (D), myocardial infarcted (MI) and diabetic myocardial infarcted (DI) rats. Data are reported as mean ± SEM

* p ≤ 0.05 compared with C; ^†^ p ≤ 0.05 compared with MI; ^‡^ p ≤ 0.05 compared with D

### Myocardial infarct size

**Fig. 2:**
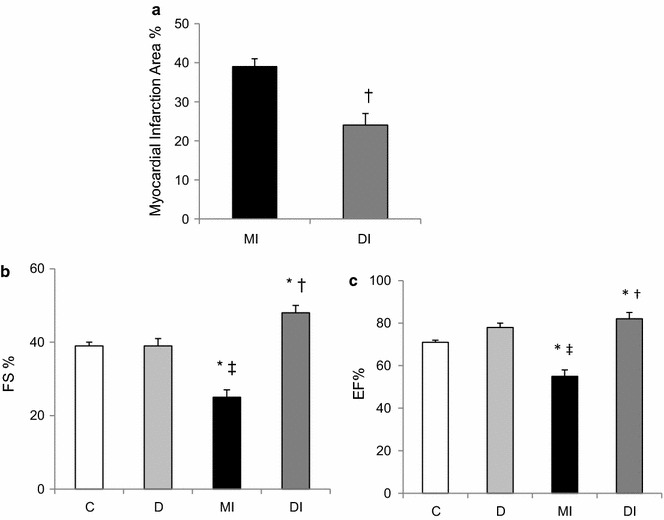
Echocardiographic parameters 15 days after myocardial infarction or after 30 days of diabetes. **a** Direct myocardial infarction area measurement in control myocardial infarcted (MI) and diabetic myocardial infarcted (DI) rats. **b** Fractional shortening and **c** Ejection fraction in normoglycemic control (C), diabetic (D), normoglycemic myocardial infarcted (MI) and diabetic myocardial infarcted (DI). Results are presented as mean ± SEM of 12 rats for each group. *p ≤ 0.05 compared with C; ^†^p ≤ 0.05 compared with MI; ^‡^p ≤ 0.05 compared with D

The myocardial infarction area of the heart was significantly smaller (24 ± 3 % of the LV perimeter) in the diabetic group when compared to the MI group (39 ± 2 % of the LV perimeter) (Fig. 2a).

### Systolic cardiac function

Systolic function, as indicated by FS (Fig. 2b) and EF (Fig. 2c), was impaired (FS: 25 ± 2 % and EF: 55 ± 3 %) in the MI group when compared to C (FS: 39 ± 1 % and EF: 71 ± 1 %) and D (FS: 39 ± 2 % and EF: 78 ± 2 %) rats. Systolic function was preserved after MI remodelling in diabetic rats (FS: 48 ± 2 % and EF: 82 ± 3 %).

### Contents of free fatty acids and triacylglycerols in the left ventricle

**Fig. 3:**
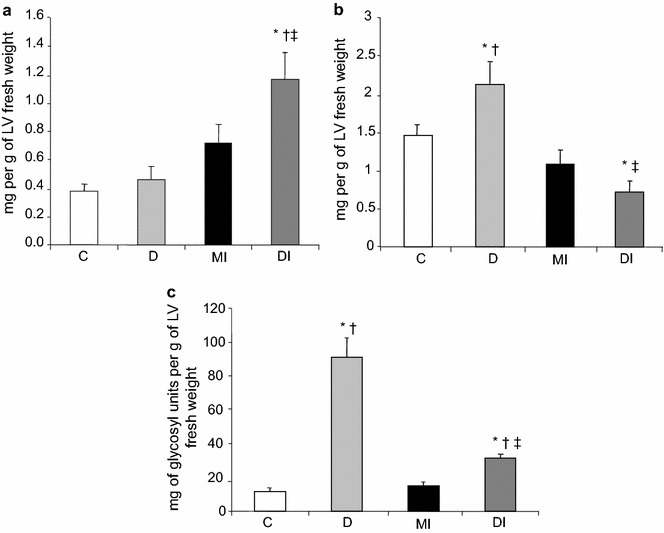
**a** Free fatty acids; **b** triacylglycerols and **c** glycogen contents in the left ventricle of normoglycemic control (C), diabetic (D), normoglycemic myocardial infarcted (MI) and diabetic myocardial infarcted (DI) rats. Results are presented as mean ± SEM of 12 rats per group. *p ≤ 0.05 compared with C; ^†^p ≤ 0.05 compared with MI; ^‡^p ≤ 0.05 compared with D

The free fatty acid levels of the LV were increased in the DI group when compared to the remaining groups (Fig. 3a). Triacylglycerol levels in the LV were increased in the D group when compared to the other groups. After myocardial infarction, triacylglycerol levels of the LV were markedly decreased in diabetic rats (DI group) but remained unaltered in the control group (Fig. 3b).

### Glycogen content in the left ventricle

Glycogen content of the LV was increased in the D group when compared to other rats (Fig. 3c). After MI tissue remodelling, glycogen content in the LV was decreased in diabetic animals but remained unaltered in the control group (Fig. 3c).

### Enzyme activities of glycolysis and Krebs cycle in the heart

**Fig. 4:**
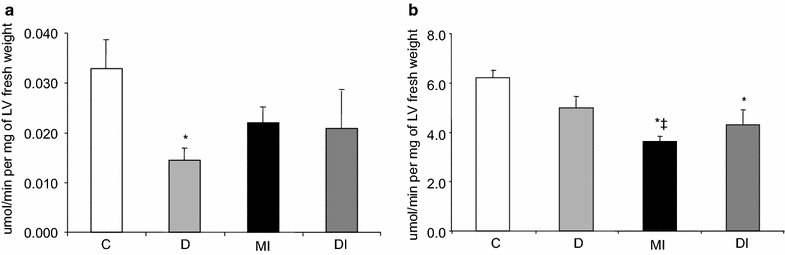
Enzyme activity in the left ventricle of normoglycemic control (C), diabetic (D), normoglycemic myocardial infarcted (MI) and diabetic myocardial infarcted (DI) rats: **a** Hexokinase and **b**. Citrate synthase. Results are presented as mean ± SEM of 12 rats per group. *p ≤ 0.05 compared with C; ^‡^p ≤ 0.05 compared with D

The maximum activity of G6PDH, phosphofructokinase, HK, and citrate synthase were measured. G6PDH and phosphofructokinase maximum activity were not markedly changed. HK activity was decreased in the heart of diabetic animals; however, after infarction and tissue remodeling (DI group), the values reached those of control rats (C and MI groups) (Fig. 4a). Citrate synthase activity was not changed by diabetes. After MI tissue remodeling, citrate synthase activity was reduced in the heart of control rats (MI versus C) but it remained unchanged in the diabetic group (DI versus D). Citrate synthase activity, however, was lower in the heart of DI when compared to C rats (Fig. 4b).

### CPT1-M expression

**Fig. 5:**
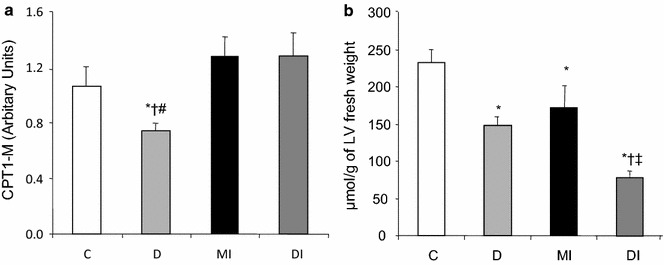
**a** CPT1-M (carnitine palmitoyltransferase I) expression and **b** ATP contents in the left ventricle of normoglycemic control (C), diabetic (D), normoglycemic myocardial infarcted (MI) and diabetic myocardial infarcted (DI) rats. Results are presented as mean ± SEM of 8 rats per group. *p ≤ 0.05 compared with C; ^†^p ≤ 0.05 compared with MI; ^#^p ≤ 0.05 compared with DI; ^‡^p ≤ 0.05 compared with D

The CPT1-M is an enzyme located in the inner mitochondrial membrane and plays a key role in the control of fatty oxidation. CPT1-M protein levels were decreased in the heart of D rats when compared to the remaining groups. Tissue remodeling abolished the decrease in CPT1-M expression observed in the heart of D rats (DI group), and values reached those of the C and MI groups (Fig. 5a).

### ATP levels in the heart

ATP levels in the heart were decreased in the D, MI, and DI groups when compared to C rats. This reduction was more pronounced in DI rats (Fig. 5b).

## Discussion

Remodeling plays a central role in cardiac tissue repair and has been associated to poor MI prognosis [17]. As previously reported by our group, diabetic rats show a smaller heart lesion area after 15 days of the MI when compared to normoglycemic animals. This reduction has been associated with decreased fibrosis and increased expression of cell survival markers, such as vascular endothelial growth factor [7], reduction in oxidative stress and attenuation of the sympathetic activity dysfunction [8]. The present study provides evidence that tissue remodeling after MI was able to preserve/recover more efficiently the systolic function in STZ-induced diabetic rats. In this sense, it should be emphasized that this experimental model of myocardial infarction allowed us to analyze the isolated effect of ischemia on the heart, regardless of the chronic vascular complications usually associated with diabetic states. Also, the jeopardizing effects of reperfusion, such as shear stress and ROS production, were prevented.

**Fig. 6:**
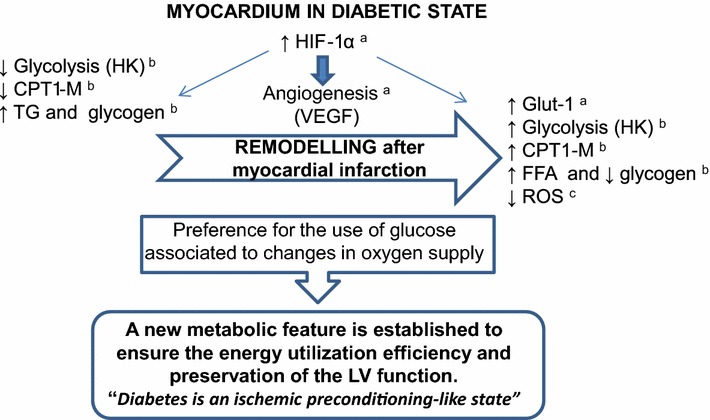
Diabetes is an ischemic preconditioning-like state by increasing the HIF-1α expression, leading to angiogenesis and modulating key metabolic pathways to optimize glucose^a^. After myocardial infarction, this heart is conditioned to increase the expression of glucose transporter 1 (Glut-1)^a^, activating glycolysis and FFA uptake (CPT1-M)^b^. The O_2_ utilization in hypoxia to generate sufficient amounts of ATP without producing excessive amounts of ROS^c^, which would inhibit tricarboxylic acid cycle and mitochondrial respiration, promotes a positive remodeling, thus preserving the left ventricular function. a [7]; b present data; c [8]

These results reported herein suggest that hyperglycemia may trigger metabolic mechanisms that would ensure better remodeling after ischemic injury. High plasma glucose levels are associated with increased expression of the hypoxia-inducible factor-1 alpha (HIF-1α). This transcription factor responds to changes in oxygen supply and regulates blood flow and glucose uptake by increasing cardiac tissue vascularization prior to ischemia [7]. These changes seem to attenuate tissue injury under repeated ischemic insults, thus favoring the consequent remodeling process. As a result, a new metabolic feature is established to ensure energy utilization efficiency and normal LV function, and an ischemic preconditioning-like state may be established in the heart from diabetic animals (Fig. 6).

Diabetic animals (D group) showed increased plasma levels of FFA and TG and raised LV levels of TG and glycogen when compared to the control group before coronary occlusion (Table 1; Fig. 3). This metabolic feature favored the remodeling of the ischemic cardiac tissue, thus preserving the systolic function. These findings suggest that in diabetic condition the infarcted heart works well after remodeling under a lower ATP level state (Fig. 5b). The mechanisms involved in the heart metabolism and function interrelationship in diabetes need further investigation; however, we might hypothesize that this association serves to enhance ATP/ADP-AMP cycling and to promote changes in angiogenesis.

We contend that the changes in glucose and fatty acid metabolism in the heart induced by diabetes are largely responsible for the results of systolic function observed.

Tissue remodeling after MI in the heart of diabetic rats somehow benefited from the metabolic feature with hyperglycemia, increased plasma levels of FFA and TG, augmented heart glycogen and triacylglycerol contents, and reduced hexokinase activity, CPT1-M expression, and ATP levels/in the heart. The metabolic profile of the heart in diabetic state involves high uptake and oxidation of fatty acids [34]. Randle et al. [35] have shown that increased plasma FFA concentrations reduce glucose utilization and causes glycogen accumulation in the heart. As previously reported, [36] free fatty acids contribute to decrease glucose oxidation in the heart after 6 weeks of streptozotocin-induced diabetes. This effect of fatty acids is partially reversed by treatment with a CPT1 inhibitor.

Hirabara et al. [37]. have shown that mitochondrial dysfunction in skeletal muscle cells treated with saturated fatty acids is associated with impaired glucose metabolism and insulin resistance, increased phosphorylation of Akt and decreased phosphorylation of GSK-3, which phosphorylates and inhibits glycogen synthase activity [38]. Insulin treatment does not change either Akt or GSK- 3 phosphorylation but increases glycogen content in the heart [39]. The reduced glycogen and triacylglycerol contents in the LV suggest that these metabolites are readily used when required by the heart. These findings may be associated with the changes in enzyme activities of glycolysis, increased FFA content, and decreased ATP levels and CPT1-M expression in the heart. The increased degradation of glycogen and TG might account for the smaller infarcted area and the preservation of the heart working capacity and systolic function found in the diabetic group. In fact, stored glycogen and TG are primary sources of energetic substrates to the heart, being particularly important in conditions of tissue injury and healing. Ranvigerova et al. [12] have shown that after one-week streptozotocin-induced diabetes, rats have higher glycogen content in the left ventricle and increased glycogenolysis activity in the heart during ischemia. As a result, the heart becomes more resistant to ischemia-induced arrhythmias in diabetes when compared to controls. Chu et al. [13] have shown reduced infarcted area and higher glycogen content in the heart of diabetic pigs.

Malfitano et al. [40] and Egorova et al. [41] have found that the increase in glucose availability, the preferred energetic substrate for the heart in stress condition, is likely to play a key cardioprotective role under ischemia. Diabetes promotes a marked increase in plasma glucose and free fatty acid levels, thus increasing the availability of these metabolites to tissues and organs, including the heart. On the other hand, the intracellular provisions of glucose (from accumulated glycogen and plasma) and FFA (from stored triacylglycerols and plasma) in the heart from diabetic rats lead cardiomyocytes to generate oxaloacetate and acetyl-CoA in order to ensure the intense flux of metabolites through the Krebs cycle. In addition to the enhanced provision of glucose and free fatty acids from the plasma and inside the cells, increases in citrate synthase activity and in CPT1-M expression were also observed in the heart from diabetic rats.

These results suggest that the heart then adapts itself to work satisfactorily in a low ATP level condition in diabetic state. This mechanism does not work in a non-diabetic state. In fact, under this latter condition, myocardial infarction remodeling was associated with a slight reduction of ATP levels together with impaired heart function. Normoglycemic rats without increased levels of TG and FFA in plasma and LV do not develop the postulated ischemic preconditioning-like state and utilize energy ineffectively [42]. In the early stages, cardiac remodeling characterized by normal or slightly increased fatty acid (FA) oxidation plays a compensatory, cardioprotective role [43], so a new metabolic profile is established to ensure energy utilization efficiency and the preservation of LV function (Fig. 6). Peroxisome proliferator-activated receptor (PPAR)-alpha is downregulated in ischemic myocardium and has been found to play a role in the switch from fatty acid oxidation to glucose consumption [44].

In conclusion, the feature of heart glucose together with free fatty acid metabolism in diabetic animals before myocardial infarction and remodeling ensure that the systolic function is preserved/recovered more efficiently when compared to normoglycemic infarcted animals. The diabetic animals presented reduced cardiac injury and a better systolic function performance associated with lower ATP levels in the left ventricle. We suggest that the diabetic state induces changes in cardiac metabolism which enhance the efficiency of tissue remodeling after ischemic injury.

### Perspectives

Several therapeutic approaches involving metabolism have been suggested to mitigate the impairment of cardiac function caused by myocardial infarction. For instance, Hughey et al. [45] have examined the efficacy of mesenchymal stem cell transplantation to attenuate the changes in cardiac function and intermediary metabolism found in myocardial infarction and insulin resistance. Evidence is presented herein that the diabetic state with hyperglycemia and increased levels of TG and FFA in plasma and heart protects may protect the heart against infarction. In fact, hypoglycemia onset as a consequence of intensive control of blood glucose levels has been associated with increased cardiovascular mortality [46–49]. Liepinsh et al. [50] have reported that the heart is better protected against myocardial infarction in the postprandial state due to high insulin-activated glucose utilization. These observations led us to speculate that the induction of a diabetic- like metabolic condition may represent a potentially novel therapeutic approach to handle non diabetic patients undergoing an ischemic heart event.

### Limitations of study

Further studies are needed to elucidate the mechanisms underlying the role played by the increased provision of glucose and free fatty acids on cardiac tissue remodeling after ischemia. One has to keep in mind that the improved cardiac function herein reported is probably transitory, lasting for a short period of time after MI. The occurrence of neuropathy and nephropathy in diabetic patients do impair cardiovascular function if hyperglycemia condition is maintained for a prolonged period of time.
